# Providers’ experience on essential health services in primary healthcare units of Ethiopia during COVID-19: a qualitative study on impact and response

**DOI:** 10.1017/S1463423624000227

**Published:** 2024-09-24

**Authors:** Getnet Mitike, Frehiwot Nigatu, Eskinder Wolka, Atkure Defar, Masresha Tessema, David Codington, Tezita Nigussie

**Affiliations:** 1 International Institute for Primary Health Care-Ethiopia, Addis Ababa, Ethiopia; 2 Ethiopian Public Health Institute, Addis Ababa, Ethiopia; 3 Johns Hopkins Bloomberg School of Public Health, International Institute for Primary Health Care-Ethiopia, Addis Ababa, Ethiopia

**Keywords:** COVID-19, Ethiopia, health services, health systems, primary health care

## Abstract

**Aim:**

The objective of this study was to explore how selected sub-national (provincial) primary healthcare units in Ethiopia responded to coronavirus disease 2019 (COVID-19) and what impact these measures had on essential health services.

**Background:**

National-level responses against the spread of COVID-19 and its consequences are well studied. However, data on capacities and challenges of sub-national health systems in mitigating the impact of COVID-19 on essential health services are limited. In countries with decentralized health systems like Ethiopia, a study of COVID-19 impacts on essential health services could inform government bodies, partners, and providers to strengthen the response against the pandemic and document lessons learned.

**Methods:**

We conducted a qualitative study, using a descriptive phenomenology research design. A total of 59 health leaders across Ethiopia’s 10 regions and 2 administrative cities were purposively selected to participate in key informant interviews. Data were collected using a semi-structured interview guide translated into a local language. Interviews were conducted in person or by phone. Coding of transcripts led to the development of categories and themes, which were finalized upon agreement between two investigators. Data were analysed using thematic analysis.

**Findings:**

Essential health services declined in the first months of the pandemic, affecting maternal and child health including deliveries, immunization, family planning services, and chronic disease services. Services declined due to patients’ and providers’ fear of contracting COVID-19, increased cost of transport, and reallocation of financial and human resources to the various activities of the response. Authorities of local governments and the health system responded to the pandemic immediately, capitalizing on multisectoral support and redirecting resources; however, the intensity of the response declined as time progressed. Future investments in health system hardware – health workers, supplies, equipment, and infrastructure as well as carefully designed interventions and coordination are needed to shore up the COVID-19 response.

## Introduction

By early January 2020, Chinese authorities had identified a novel coronavirus causing a ‘pneumonia of unknown origin’ (Chen *et al.*, 2020). A few weeks later, the World Health Organization declared the virus a ‘public health emergency of international concern’, calling it severe acute respiratory syndrome coronavirus-2 (SARS-CoV-2) (WHO, 2020a, 2020b).

COVID-19 has a multifaceted impact on the health systems of Africa (African Union, 2020; Tessema *et al.*, 2021). As a result, its impact may stagnate or reverse the improvement of the decades of investment in primary health care (PHC) in maternal and child health and communicable diseases.

Ethiopia’s preparatory activities were challenged after the first cases of coronavirus disease 2019 (COVID-19) were reported on 13 March 2020 (WHO, 2020c). Screening at airports and acquisition of equipment, including test kits, face masks, and protective wear, were followed by a state of emergency that shut off facilities such as schools and universities. State actions also limited gatherings and number of passengers in transportation services. People were obliged to wear masks and use sanitizers in public places (PERC, 2020; Mohammed *et al.*, 2020). Education and preventive strategies were continuously disseminated, and priority was given to equip and expand public and private laboratories for COVID testing (Kebede *et al.*, 2021).

Early reports indicated COVID-19 has impacted essential health services. Furthermore, learning from past epidemics the potential for secondary effects such as disruption of health services that get worse in the process was underscored (Elston *et al.*, 2017; PERC Ethiopia, 2020). Data on the impact of the COVID-19 pandemic on essential health services were scarce, particularly at sub-national and facility levels in Ethiopia. This study was conducted to understand how the Ethiopian PHC structures fared during the first phase of the COVID-19 pandemic with the aim to explore impacts on essential health services. Key informant interviews were held with healthcare leaders, providers, and managers at lower levels of the health system that faced day-to-day challenges.

## Methods

### Study setting

The study was conducted in 10 regions of Ethiopia by involving regional health bureaus, zonal health departments, woreda (district) health offices, and health providers at facilities. Ethiopia follows a federal system where regions and districts (woredas) have councils that make decisions at the local level. Regions are subdivided into zones or sub-cities in the case of Addis Ababa, and zones are further divided into woredas (districts) where facilities are located. Regional health bureaus are prototypes of the Ministry of Health (MOH) at the regional level. Because of the decentralization, regions and woredas are accountable to local governments. Facilities (except hospitals) at the woreda level (health centres with health posts and primary hospitals) report to the woreda (district) health offices and form functional units called PHC units. Ethiopia is commended for its flagship PHC programme known as the ‘Health Extension Programme’ that was credited for achieving the Millennium Development Goals such as reducing the child mortality rate and reduction of the maternal mortality ratio close to the target. It also contributed to the achievements in tuberculosis (TB), human immunodeficiency virus (HIV), and malaria programmes. The Health Extension Programme required the highest political backing at inception as well as its expansion and implementation.

### Design

A qualitative study with a phenomenology design was used. Key informant interviews were conducted to explore the impact on essential health services at the sub-national level in Ethiopia covering the first six months into the COVID-19 pandemic. We followed the Consolidated Criteria for Qualitative Studies (COREQ) guideline to write the report (Tong *et al*., 2007).

### Selection of participants and sample size

Fifty-nine key informants participated in the study. We used purposive sampling to select the participants. Key informants were chosen from regional health bureaus, zonal health departments, and health facilities. We selected five participants from each region, that is, from a regional health bureau (one), from zonal/woreda health offices (two), and from health facilities (two). We assumed that the inclusion of all regions would result in a more robust data set due to variations of capacities in the study areas, and hence, we prioritized coverage over the application of saturation.

### Data collection and analysis

Twelve interviewers and 12 supervisors collected the data. All had graduate-level education with experience in qualitative research and corresponding requirements such as probing and asking open-ended questions. The training was conducted remotely via Google Meet for half a day due to COVID-19 restrictions and travel bans. The discussion included question-by-question reviews with both Amharic and English versions. Since the data collectors and supervisors were office-holders in the same area, it was believed that they were similar to the respondents in many ways. Data was collected using a semi-structured guide that was translated into the local language (Amharic) which is widely spoken by health professionals. The guide was developed from the latest evidence on COVID-19 at the time of the study. Interviews took place in person or by phone when not possible. In the case of face-to-face interviews, offices of regional health bureaus, zones, and health facilities were used. These allowed privacy for the interviews. Data collectors digitally recorded the interviews and transcribed and translated them into English. For this study, essential health services are defined as stated by the MOH of Ethiopia, that is, ‘all promotive, preventive, curative and rehabilitative interventions relevant for low- and middle-income countries including but not limited to communicable diseases, maternal health, child health, NCDs, injuries, surgery and neglected tropical diseases (NTDs). It also considered other system-wide interventions, such as health education, and communications, laws and regulations’ (MOH, 2019).

Data analysis was conducted by the PI (GK) and the co-PI (TN) independently. GK is an MD with a PhD in public health and has extensive experience in qualitative research. TN has graduate-level training in social work and public health with expertise in qualitative studies. Both are experienced in designing, conducting, and analysing qualitative studies. The investigators did not have any direct contact with study participants, and the data were collected by experts in the respective regions. All the other investigators had a range of experience in qualitative studies. As part of the data analysis: First, the transcripts were read and reread and were coded independently by the two investigators. Then, the codes were developed both manually and electronically (using NVIVO). Second, we agreed on the list of codes (both a priori from the guideline and from the data), and a codebook was prepared and revised to accommodate changes, modifications, and additions that emerged through the process of the coding and analysis. Third, thematic analysis was used to generate themes and subthemes from the codes and categories. Thematic analysis was used because of its relative advantage of flexibility in terms of allowing detailed descriptions (thick) of the data (Braun and Clarke, 2006).

To ensure the credibility of our findings, we recruited data collectors who were actively working in and familiar with the respective study areas and the health system. The data collectors also received training on the purpose, methods, and tools of the study. We accounted for confirmability through recordings and transcriptions that were reread and checked by investigators. To strengthen dependability, we have included the interview guides in the annex of the report so that they may inform researchers in other settings for similar endeavours. The study addressed transferability by covering all regions of the country to capture possible contextual differences.

*Ethical considerations:* This study was approved by the Institutional Review Board of the Ethiopian Public Health Institute (EPHI). Respondents were informed about the study, and their consent was obtained. The data collectors took appropriate protective measures in line with the guidelines for the prevention of COVID-19. Names of respondents were not included in the data collection except for their sociodemographic variables such as sex, age, qualification, and number of service years. While reporting the data, personal identifiers were not used.

## Results

### Study participants

Out of 60 potential respondents, 59 participated in the study. The mean age of participants was 35 years, and the overwhelming majority (89.9%) were male. Nearly half (47.5%) of the participants held a baccalaureate degree (health officers, nurses, and other degrees), and 44% were providers in health facilities. Their service years ranged from 2 to 40 years.

Based on the analysis, the result is organized using the following themes and subthemes: (1) impact on essential health services (with five subthemes: impact on service utilization and service delivery, services affected by COVID-19, impact on health outcomes, impact on the health workforce, and measures taken to maintain essential health services) and (2) participants’ assessment of COVID-19 response and challenges (with two subthemes: participants’ assessment of the response to COVID-19 and challenges).

### Theme 1: impact on essential health services

#### Impact on service utilization and service delivery

Study participants reported that the utilization of essential health services considerably declined in the first few months after COVID-19 was detected in Ethiopia. A regional health bureau official in Amhara estimated a one-fifth reduction in utilization in his region. In Southern Nations and Nationalities Peoples’ Region (SNNPR), a regional health bureau representative also reported that family planning users had declined by about 200 000 and that first and fourth antenatal care visits had dropped by between 10 000 and 15 000 attendants. Interviewees attributed these changes to a decline in care seeking, in service delivery, or both. They identified fear of infection as the key factor that kept patients from seeking care, particularly among the chronically ill. According to a zonal health official in Amhara, health workers even encouraged high-risk groups, such as HIV/AIDS patients, to stay home. Compounding this, real or rumoured cases of COVID-19 infection among care providers fuelled people’s reluctance to visit health facilities. In the SNNP and Tigray regions, the following responses were obtained regarding outpatient and chronic care services, respectively:

*Since we focused on prevention and management of COVID-19, it has affected the delivery of essential health services. For instance, in our health centre, we had more than 400 cases of hypertension on follow up. After the introduction of COVID-19, we lost many of these patients. The reason for this is fear. They are aware that COVID-19 is more severe in individuals with known chronic diseases like hypertension. Similarly, we have lost many HIV positive patients to follow-up. The other major reason for the reduction in client flow, especially among those with regular follow-up, is the lockdown imposed by the command post.*

(TG_HF_1)

Although there was not a complete lockdown at the national level, Ethiopia introduced a state of emergency between March and September 2020. According to several key informants, limited transportation options due to the state of emergency resulted in increased passenger fees impacting patients and health workers in need of follow-up care and women in labour. The state of emergency also led to business closures and job losses that affected people’s ability to access health services. The possibility of being tested and quarantined served as another reason for many patients and community members who might otherwise have sought care.

*It limited community mobility and transportation cost was doubled. So, people are not coming to health facilities for seeking care. The income of people also declined. For example, hotel employees lost their jobs, tea and coffee owners were banned from providing services for some time, etc. which affected the income of people in turn with impact on health services*.

(SN_HF_ 2)

Key informants described that, when the pandemic occurred, they neglected other priorities to focus almost exclusively on COVID-19:

*The concern of the leaders was that we will lose lives due to coronavirus. Corona will kill all. So, we have to focus only on corona: no agriculture, no peace and security, and even no politics*.

(AF_RHB_2)

Financial, human, and material resources were redirected towards the pandemic. This shift in focus affected the availability of health workers. Many health workers were reassigned to the COVID-19 response effort, and finances allocated for essential health services were diverted to purchase personal protective equipment (PPE).

Study participants also reported that health providers and health extension workers stayed home in the early days of the pandemic, fearing infection. Like other community members, health workers were affected by constraints on public transportation imposed by the state of emergency and increases in transportation fees.

Furthermore, many health facilities faced a shortage of essential drugs and supplies, particularly vaccines, anti-retroviral drugs, and HIV test kits. Due to international lockdowns, the Ethiopian Pharmaceutical Supply Agency – the major public supplier of essential drugs to public health institutions – struggled to meet supply needs. Health facilities that could draw from internal revenues were forced to buy from the private market but at a very high cost.

*… When the condition became worse, all the suppliers doubled the price of basic medical resources. You will be surprised; the medical suppliers in the private sector raised their prices 10- to 15-fold compared with the government suppliers….*

(AM_HF_2)

Due to a combination of these factors, many health facilities stopped or limited their services in the early months of the pandemic. Some provided only emergency or critical care, others offered services only for chronically ill patients, and a few kept all services functioning except major operations.

On the other hand, a few participants reported no changes in service utilization or delivery, and in a few, even an increase was observed. In Sidama, Amhara, SNNP, and Tigray, woreda and health centre leaders witnessed an increase in patient flow. They explained that some health facilities remained open while others were temporarily shut down shifting the patient load. As a result, patients migrated to the health centres that were offering services at the time. Health centres also absorbed new patients when hospitals were converted to treatment or isolation centres. Patients sought alternative sources of care even after hospitals reverted to routine services because they perceived that the risk of exposure in hospitals was greater than in health centres. In some cases, parts of hospitals were closed for providing care to patients with COVID-19 adding to their fear of transmission to other clients.

*…Most of the clients, even after the hospital corrected the first direction, perceived that health centres are relatively less exposed to COVID than the referral hospital. If you take Maraki Health Centre for example, it served 69,000 outpatients, Azezo Health Centre had 40,000 outpatients. These figures are very high compared with projections for their catchment areas.*

(AM_HF_1)

#### Services affected by COVID-19

The services most affected by the decline in service utilization and delivery were HIV/AIDS, immunization, institutional delivery, antenatal care, family planning, TB, and child nutrition. Many HIV/AIDS patients were lost to follow-up and interrupted their care due to fear of infection:

*From the 9000 ART clients who were using our facilities, nearly one-third, dropped their services*.

(AM_ZHD)

Parents were afraid to bring their children for immunization, and some facilities stopped their immunization services for a period. Women in labour faced a similar combination of fear and curtailed options for care.

*…for example, we used to admit malnourished children. There will be many defaulters among them because we don’t admit. … The other one is ANC, ANC-4 has declined. Mothers used to come to the health centre starting in their first term until about one week before their deliveries. They used to stay in the maternal waiting room and deliver here. That has declined. And even family planning. These are the three affected service areas.*

(AF_HF_1)

#### Impact on health outcomes

The decline in essential health service utilization and delivery profoundly impacted women and children. Many participants spoke about an increase in home births, which had previously been on a downward trend, and linked home births to maternal and newborn deaths. Health officials in Afar, Tigray, and Amhara witnessed an outbreak of measles due to interruptions in the vaccination programme, and officials in Sidama and Harari saw a rise in severe acute malnutrition.

*It is difficult to talk about the number and severity in the absence of a study. But I can tell you what I know. For example, a mother called for an ambulance service after she gave birth at home … After a ruptured placenta, she arrived at our centre with post-partum haemorrhage and we referred her to Hawassa Reference Hospital, where she died. The Hawassa city administration and Millennium Health Centre have information about maternal deaths. There were no maternal deaths recorded in the last two years. But after COVID was detected in our city, we registered the case that I just mentioned. Even though we cannot describe the consequence in numbers, because we don’t have the data, we know there are unnecessary deaths.*

(SID_HF_1)

#### Impact on the health workforce

Redeploying health professionals affected the delivery of essential health services. According to a regional health bureau official in Benishangul-Gumuz, ‘As soon as COVID-19 occurred, by providing training, we mobilized the health workforce that was previously providing essential health services’. New staff were rarely hired to replace the ones who were assigned to work on COVID-19, and as a result, routine services were interrupted, and quality was compromised in the early months of the pandemic.

*When we look at human resources, after the introduction of coronavirus, four or five staff were moved from the health centre to screening and treatment centres. So, this was challenging for us. Reducing our workforce by four or five people at a time of high workload was really challenging. Despite this fact, there were no new staff recruited by the health bureau.*

(TG_ HF_2)

#### Measures taken to maintain essential health services

A few months into the pandemic, the danger of neglecting essential services became clear.

*Then after about three months, by assessing the impact of COVID-19 on essential health services, we found that death from non-COVID morbidities was greater than COVID-19 deaths. Therefore, a guideline was developed to work on both COVID and non-COVID activities, side by side.*

(BG_RHB)

The MOH issued a guideline to maintain essential health services in the context of the pandemic. Following the guideline, health offices across the health system divided their planning and implementation teams into two: one that focused on COVID-19 (FMOH Management Handbook, 2020) and another that focused on essential health services (FMOH Implementation Guide, 2020). Health workers returned to regular posts, or new staff were hired to replace them. Health extension workers mobilized community members, provided health education, and tracked down patients who had missed appointments. A new protocol was issued and implemented that allowed for TB and HIV/AIDS patients to receive prescriptions that lasted three to four months protecting them from frequent exposures.

### Theme 2: participants’ assessment of the response to COVID-19 and challenges

#### Participants’ assessment of the response to COVID-19

When assessing the health system’s response to COVID-19, key informants stressed the commitment of staff across the system, their sense of common purpose, and the spirit of collaboration across sectors. They cited the willingness of staff, especially at lower levels, to do whatever was asked of them. They gave examples of health professionals working 24 h a day, without additional compensation and with limited resources. In their determination to protect communities from infection, key informants noted that hierarchies broke down and high-ranking officials worked alongside with health extension workers to create awareness about the virus on the streets and in markets.

The respondents also stressed the important work accomplished through multisectoral partnerships. Academic institutions and the education sector, non-governmental organizations, businesses, security forces, and private citizens aided the response effort. Schools offered space for isolation and treatment centres. Academics advised coordination committees, especially at regional levels, and participated in community education initiatives. Non-governmental organizations helped organize trainings and supplied the health system with PPE when needed. And the police provided protection for and support to health extension workers as they conducted house-to-house screening and education and enforced prevention measures in public spaces.

However, the study participants perceived that the health system’s preparation for and response to the pandemic was inadequate, and hence, the impact on essential health services was noticeable. The majority pointed out that the health system did not have the necessary budget, infrastructure, supplies and equipment, and workforce to respond properly and quickly to a global pandemic of such magnitude. The budget problems affected not only their ability to purchase needed items but also to compensate health workers for the overtime pay that they were promised by authorities. Their criticism of the system was compounded by their perception of poor leadership that failed to mobilize sufficient resources to be responsive to requests from lower structures.

*…our preparation was not satisfactory. The readiness of health facilities on prevention and response was not satisfactory. Health facilities were not well equipped with drugs, supplies, and health care providers. These are among the weaknesses.*

(SN_RHB)

#### Challenges

Study participants identified resource limitations as the major barrier to ongoing COVID-19 response. They also stressed the inability of the health system to function properly without adequate budget, supplies, equipment, and workforce. There were major concerns over the health system’s capacity to maintain essential health services in the context of a worsening pandemic, experiencing additional health emergencies (cholera, malaria, and measles), and environmental disasters affecting different parts of the country.

Along with the structural and supply-side challenges, factors affecting communities’ response were emerging. For instance, participants pointed out a worrisome rise in community reluctance to observe prevention measures, particularly wearing masks. Community members had begun to doubt the seriousness of the pandemic as they witnessed few severe cases and deaths. People in some regions saw the pandemic as a ploy by the federal government to postpone elections. Health officials also feared that the end of the state of emergency gave community members the impression that the pandemic was no longer a threat.

*The main challenge is attitude. People assume that COVID-19 is political. Some believe health professionals are earning special fees. Some say you are earning 35,000 birr monthly. They believe that health professionals are working for their money and per diem. These are the challenges. Nowadays, a great challenge is the ending of the state of emergency. Now people are saying there is no COVID-19 anymore. Previously, transportation was at half capacity, you were not allowed to walk out without a face mask, social distancing was mandatory, etc. But when the state of emergency was lifted off, there was no new direction set. Then people are not trusting health professionals, they are telling us that COVID does not exist. This type of attitude is a great challenge.*

(SN_WoHO)

Study participants suggested improving the response by addressing the structural problems that keep the health system weak, controlling the spread of COVID-19 – especially by investing in PPE for staff – improving surveillance, testing capacity, and strengthening community education.

## Discussion

In this study, we identified many ways in which the COVID-19 pandemic impacted service delivery and utilization as part of PHC in Ethiopia.

The most important finding of our study is the decline in service delivery across Ethiopia due to the reallocation of health systems resources, decline in health workforce, and shortages of essential drugs and supplies, affecting HIV/AIDS and TB services, immunizations, and maternal and child healthcare services. Not only did the reallocation of resources affect the provision of essential services, but higher-level measures such as national and international lockdowns restricted transportation and supply chains, affecting services.

Ethiopia has put in place appropriate guidelines and policies (MOH, 2019; MOH, 2020) to combat the spread of COVID-19 and mitigate its impact. However, the multifaceted impact of the pandemic combined with the limited capacity of the system has brought serious challenges to the provision of health services. Previous research from Ethiopia and other African countries has similarly shown that the provision of essential services declined during the early months of the COVID-19 pandemic, as well as during other epidemics (Tessema *et al.*, 2021). A review of registers at a referral hospital in the Amhara region of Ethiopia found a 50% decline in antenatal care, a 70% decline in antenatal and childhood emergency visits, and a 95% decline in family planning visits during the implementation of COVID-19 control measures in the early months of 2020 (Abdela *et al.*, 2020). Studies from Kenya, Nigeria, and South Africa also showed reductions in antenatal care, immunization, and family planning utilization (Kimani *et al.*, 2020; Ahmed *et al.*, 2021). Similar to our findings, the studies attributed the effects to restrictions in movement imposed by the state of emergency and curfews as well as to fear of infection by community members (Kimani *et al.*, 2020; Ahmed *et al.*, 2021). In Guinea, a study on the effects of the 2014 West African Ebola epidemic on health services attributed a decline in service provision to healthcare workers’ fear of infection and transportation barriers (Delamou *et al.*, 2017).

The second most important finding is the decline in service utilization due to fear of infection, transportation barriers such as high fees and emergency closures, and fewer options for care. Our study found that the patients likely to stop accessing services were those who were most vulnerable, such as the chronically ill, the immunocompromised, and those unable to access transportation. This should not be surprising, as previous research has shown that secondary effects of disease outbreaks and disasters on health systems disproportionately affect people who are vulnerable. United Nations Population Fund (UNFPA) technical report on reproductive, maternal, newborn, and adolescent health during pandemics highlighted the particular vulnerability of women and children not just during the 2013–2016 Ebola outbreak in West Africa but also during the early months of COVID-19 (UNFPA WCARO, 2020). The Ebola outbreak induced a decline in access to services that was experienced mostly by pregnant women, children, the elderly, and those with chronic illnesses (Elston *et al.*, 2017).

The combined reduction in both service delivery and utilization was reflected in health facility data as well as indications of rising morbidities and mortalities in the community. The disruption of services aggravated the burden of diseases. A measles vaccination programme was delayed, resulting in an outbreak of measles, and severe acute malnutrition became more prevalent in some areas. These trends primarily impacted women and children. However, one should interpret the findings cautiously as our study had not covered beneficiaries of the services who could have provided some additional perspectives.

A few months into the pandemic, the MOH addressed the decline in essential health services by issuing policies to maintain these services while attempting to protect those with vulnerabilities (MOH, 2020). Policies to maintain essential health services were commonly issued worldwide in response to the pandemic, as demonstrated by a questionnaire the World Health Organization (WHO) issued to MOH officials in 159 countries in May 2020. Of the 105 countries that responded, 66% had created policies to maintain a core set of essential services during the pandemic, and countries that had already identified a package of essential services prior to the pandemic were more likely to create policies to maintain these alongside a COVID-19 response (WHO, 2020d). According to the survey, services were disrupted on both the supply side, such as elective procedures being cancelled, and the demand side, as lockdowns restricted patients’ access to facilities. This points to the importance of quick action on the part of health officials and policymakers to address the pandemic as well as disruption to essential services. Yet even as responsive policies and measures are set in place, health systems recovery often isn’t immediate, and reinstallation of services doesn’t necessarily mean recovery of lost health gains. A third survey round in late 2021 by the WHO on essential health services showed that while services recovered to some extent in most countries, the vast majority of countries still had some disruption to essential services (WHO, 2022).

Our study participants rated the response effort highly, especially at the beginning of the pandemic, and stressed the intensity of focus and common sense of purpose. But as the pandemic progressed, they witnessed a decline in commitment, especially among partners in other sectors, and a resistance to prevention measures on the part of the public. Most of all, they expressed concern that a weak health system that lacked adequate funding, human resources, infrastructure, and supplies and equipment could not adequately respond to the dual responsibilities of fighting a global pandemic and maintaining essential health services.

A strength of this study is that it included perspectives from health providers, health officials, and policymakers from all regions of Ethiopia. The data collectors were familiar with the health system and its context which made it possible to contact participants and conduct the interviews. A limitation of this study is the lack of a granular quantitative description of the response efforts to the pandemic, particularly regarding limitations in budget, infrastructure, supplies, and workforce. Additionally, the findings may not adequately represent the local COVID-19 response across all facilities and regions of Ethiopia. Furthermore, the fact that we did not include the experiences of beneficiaries and other stakeholders that are key to Ethiopia’s response to the pandemic including government officials and implementing partners would have some implications.

## Conclusion

Local health systems were unable to maintain essential health services during the early phases of the pandemic, potentially derailing progress that Ethiopia has made over decades in maternal and child health and communicable diseases such as HIV/AIDS and TB. As the number of infected people in Ethiopia rises, we recommend reengaging local administrations and actors outside the health sector, including academic institutions and local businesses, in the response effort, strengthening resource mobilization for COVID supplies and equipment, improving testing capacity across the country, and collaborating with local media and community leaders to dispel public misconceptions about COVID-19 and strengthen community adherence to prevention measures. We recommend that health officials and administrators seek solutions tailored to their facilities and local structures to increase local supplies of drugs and other supplies essential to health facility functioning. Epidemic preparedness in the future should seek measures to maintain essential health services parallel to crisis response from the very start, with a particular focus on providing access to the most vulnerable. Policy-level preparations for engaging the private sector through advocacy, strengthening public-private partnership, and putting in place regulatory mechanisms would be helpful. Additionally, there remains a need for mixed methods research to better understand beneficiaries’ perspective of impacts on the services and learn from the strengths and weaknesses of Ethiopia’s health system response to COVID-19 to build capacity and enhance preparedness for future health emergencies. In a broader perspective, the results of this study could serve as a lesson to other low-or middle-income countries in order to develop strategies to better cope with the health situation in the context of the COVID-19 pandemic.
